# Volume-Based Care among Young Women Diagnosed with Uterine Cancer

**DOI:** 10.5402/2011/541461

**Published:** 2011-11-24

**Authors:** Teresa P. Diaz-Montes, Robert L. Giuntoli

**Affiliations:** Kelly Gynecologic Oncology Service, Department of Gynecology and Obstetrics, Johns Hopkins Medical Institutions, 600 North Wolfe Street, Phipps #281, Baltimore, MD 21287, USA

## Abstract

*Purpose*. To characterize volume-based care of uterine cancer among women aged ≤50 years. *Methods*. The Maryland Health Service Cost Review Commission database was accessed for uterine cancer surgical cases from 1994 to 2005. Cross-tabulations and logistic regression models were used to evaluate for significant associations among volume-based care and other variables comparing women ≤50 years with those aged >50 years. *Results*. Women ≤50 years comprised 13.6% of the cases. Women ≤50 years were less likely to be managed by high-volume surgeons (31.6% versus 35.1%, *P* = 0.02). For women ≤50 years, there was a trend toward management at low-volume hospitals (52.0% versus 54.0%, *P* = 0.22). No deaths were reported among the group of women ≤50 years treated by high-volume providers or at high-volume centers. Women ≤50 years managed by high-volume surgeons had longer length of stay (*P* < 0.001) and higher adjusted cost of hospital-related care (*P* < 0.00). Women ≤50 years managed at high-volume centers had higher adjusted cost of hospital-related care (*P* = 0.01). *Conclusion*. Primary surgical care of young women with uterine cancer is often performed by low-volume providers.

## 1. Introduction

Uterine cancer is the most common diagnosed gynecologic cancer among women in the United States [1], with 43,470 estimated new cases and approximately 7,950 estimated deaths in 2010 [2]. Although uterine cancer is found mainly in the older patient population, up to 14% of uterine cancers are found in women younger than 45 years of age [3]. Most studies have demonstrated that younger women have a lower risk of death from uterine cancer than older women independent of stage at diagnosis [4, 5]. Surgical management of uterine cancer entails a staging procedure which includes a total hysterectomy, salpingooophorectomy, and possible lymph node dissection depending on the spread of the disease and histological type. 

Postmenopausal and abnormal premenopausal bleeding are the primary symptoms of uterine cancer. A high index of suspicion must be maintained if the diagnosis of uterine cancer is to be made in the young patient. Young women with or without risk factors may present with abnormalities in their menstrual periods, which can be initially treated with hormonal therapy, thereby delaying the diagnosis of cancer. Some risk factors for uterine malignancy include prolonged exposure to unopposed estrogen either endogenous, (e.g., early menarche, late menopause, nulliparity, polycystic ovarian syndrome, obesity), or exogenous (e.g., estrogen therapy or hormone replacement without progesterone) [3]. Other independent risk factors include diabetes and hypertension [3]. Uterine cancer in young women is often associated with early-stage disease, well-differentiated tumors, and a good prognosis [6–8].

The actual care delivered to women diagnosed with uterine cancer appears to depend upon a number of variables including surgeon training, hospital volume, extent of disease, patient characteristics, and physician access. These women are mostly managed by gynecologic oncologists as well as by nongynecologic oncologists (general gynecologists and general surgeons). Gynecologic oncologists are specifically trained to perform the required staging and cytoreductive surgical procedures for uterine cancer. However, patterns of care studies have shown that gynecologic oncologists provided care in 48.8% of the patients and gynecologists in 50.2% of the cases [9]. General surgeons assisted gynecologists in 36.5% of cases [9]. Roland et al. [9] have shown that the gynecologic oncologist completed the surgical staging two times more frequently than the general gynecologist (94.0% versus 45.2%, *P* < 0.05). They concluded that women with uterine cancer managed by gynecologic oncologists are more likely to receive comprehensive surgical staging resulting in an efficient use of health care resources and minimizing the potential morbidity associated with adjuvant radiation therapy [9].

Numerous studies have evaluated the association of surgeon case volume with clinical outcomes for various procedures and have shown that higher surgeon volume is associated with improved outcomes [10–13]. While specialty training is critically important to quality cancer care, recent attention has also focused on the positive relationship between surgeon and hospital case volume and clinical outcomes for malignancies treated with technically complex surgical procedures [14]. Recently, there has been an interest in elucidating patterns of care for patients treated with uterine cancer. Most studies evaluating age-based outcomes for uterine cancer have been single institution analysis containing only a small portion of patients. Age-based analysis of resource allocation has not been performed for younger patients. More specifically, prior studies have not evaluated whether care provided by high-volume surgeons at high-volume centers leads to differences in resource allocation and short-term survival among younger patients. Therefore, the goals of this study were to evaluate volume-based care for women with uterine cancer aged 50 years and younger using a statewide population database.

## 2. Methods

Approval to conduct this study was obtained from the Johns Hopkins Medical Institutions Clinical Research Committee and Joint Committee on Clinical Investigation, and the requirements for informed patient consent were waived. The study design was a cross-sectional analysis of hospital discharge data from the nonfederal acute care hospitals in Maryland collected by the Maryland Health Services Cost Review Committee (HSCRC). The HSCRC database provides information regarding the index hospital admissions and is limited to 30 days of followup. The main study objective was to characterize volume-based care among women ≤50 years when compared to women >50 years. A subgroup analysis among women ≤50 years with regards to surgeon and hospital uterine cancer volume was performed to evaluate the risk of in-hospital related death and associations with the length of hospital stay, hospital-related costs, and ICU length of stay.

All adult female patients, 18 years of age and older, who underwent a surgical procedure including a hysterectomy for a malignant uterine neoplasm in Maryland between January 1, 1994 until December 31, 2005 were included in the study. The International Classification of Disease, 9th revision (ICD-9) code 182.0 (malignant uterine neoplasm) was used for sorting. All histologic types of uterine cancer were included in the search. The surgical procedures included in the analysis were limited to those incorporating hysterectomy as this was felt to be the most likely to capture those patients undergoing initial surgery for uterine cancer.

The main independent variable was patients' age. Patient age was modeled as a categorical variable (patients 50 years of age and younger compared to patients over 50 years of age). This age cutoff was designated based on the average age of menopause as 51.4 years [15, 16]. Frequency distribution for variables like ethnicity, insurance payer status, hospital volume, surgeon volume, hospital type, inpatient death, and concordance between attending and operating physician was tabulated among the two groups. Information regarding International Federation of Gynecology and Obstetrics (FIGO) or American Joint Committee on Cancer (AJCC) stage of disease, tumor grade, histological subtype, extent of disease, or residual disease was not available from the HSCRC database. In addition, the HSCRC database only provides clinical information for the index hospital admission, so that data on clinical outcomes beyond this time period could not be assessed. Both surgeon and hospital volume were modeled as categorical variables. Based on previous research in volume-based care in uterine cancer, surgeons performing ≥100 cases of uterine cancer per study period were categorized as high volume, and those performing <100 cases of uterine cancer per study period as low volume [17]. Surgeons were included in the analysis if they performed at least one uterine cancer surgery during the entire study period. Based on previous research in volume-based care in uterine cancer, hospitals with ≥200 cases of uterine cancer per study period were categorized as high volume, while those with <200 cases of uterine cancer per study period were categorized as low volume [17]. Similarly, hospitals were included in the analysis if at least one uterine cancer surgery was performed during the entire study period. A community teaching hospital was defined as a nonuniversity hospital with a residency program in Obstetrics and Gynecology. Hospital-related charges for each index admission were converted to the organizational cost of providing care using cost to charge ratios for individual hospitals. Cost to charge ratios were calculated from data from the Health Services Cost Review Commission by dividing the average inpatient expense by the average inpatient revenue of each hospital during each year of the study interval [18]. This ratio was then multiplied by each patients' charge to obtain the cost per admission [19]. All costs were converted to 2005 USD [20].

Cross-tabulations were analyzed using chi-square tests. *T*-test for simple linear regression was employed to evaluate adjusted cost of hospital-related care, intensive care unit length of stay, and admission length of stay among the two age groups. *T*-test for simple linear regression was done for the subset group of women aged 50 years and younger to evaluate the variables of adjusted cost of hospital-related care, admission length of stay, and intensive care unit length of stay among hospital's and surgeon's volume. All statistical computations were performed using the SAS system [21], and all reported *P* values are two sided.

## 3. Results

A total of 6,181 women who met the criteria for a primary surgical procedure for a malignant uterine neoplasm were identified in the state of Maryland during the period of 1994–2005. Women aged 50 years and younger comprised 13.6% (*n* = 844) of the cases. Most of the women aged 50 years and younger were white (78.0%), had insurance coverage through an HMO (40.3%), and were treated in a community-based hospital (67.4%) (Table 1). Young women were more likely than older women to have concordance between the operating physician and the attending physician (*P* = 0.03) (Table 1). In other words, young women were more likely to have an operating surgeon that was the same clinician of record. Younger women were more likely to have shorter length of hospitalization stay (*P* < 0.0001) and lower adjusted cost of hospital-related care (*P* = 0.0002). There were no significant differences between the lengths of intensive care stay between groups (Table 2). Overall, 85 deaths were reported during the immediate 30-day period. Four of the reported deaths occur among the group of women 50 years of age and younger. Information regarding the cause of death was not available from the database. 

In total, 894 different surgeons provided primary uterine cancer surgical care, although not all surgeons provided continuous care for the entire duration of the study period. Only 9 (1.0%) of the surgeons were categorized as high-volume surgeons. Women aged 50 years and younger were less likely to be managed by high-volume surgeons when compared to women older than 50 years (31.6% versus 35.1%, resp., *P* = 0.02). In the subgroup of women aged 50 years and younger, those that were managed by high-volume surgeons were more likely to have longer hospitalization stays (*P* = 0.003) and higher adjusted cost of hospital-related care (*P* < 0.0001) (Table 3). No difference was observed in terms of ICU length of stay between high- and low-volume surgeons. No deaths were reported among high-volume surgeons during the immediate 30-day period. Due to the lack of events on one of the groups, a statistical comparison of death rates between high- and low-volume surgeons could not be performed.

A total of 49 hospitals provided care for uterine cancer patients during the study period. Only 8 (16.3%) hospitals meet the criteria of high-volume hospitals. No difference was observed among groups in terms of access to high-volume centers (52.0% versus 54.0%, *P* = 0.22). In the subgroup of women 50 years of age and younger, those admitted to a high-volume hospital were more likely to have higher adjusted cost of hospital-related care (*P* = 0.01) (Table 4). No difference was observed among hospital and ICU length of stay among groups. No deaths were reported among high-volume hospitals during the immediate 30-day period. Due to the lack of events on one of the groups, a statistical comparison of death rates between high- and low-volume surgeons could not be performed.

## 4. Discussion

Approximately 14% of uterine cancers develop among women that are younger than 45 years [3]. Several studies have demonstrated that age is an independent predictor of overall survival [4, 5]. Our analysis of the HSCRC database from 1994–2005 confirms that younger women have lower short-term mortality than older women diagnosed with uterine cancer. The fact that younger women have better outcomes can be explained by a number of hypotheses. It may be that younger individuals have a better health profile, with less morbidity and concurrent medical problems. Younger body/tissues may respond to a surgical insult with less postoperative complications and accelerated healing. Also, uterine cancer in young women is often associated with early-stage disease, well-differentiated tumors, and a good prognosis [6–8]. Even though the lack of events did not allow performing a statistical analysis, no in-hospital related deaths were reported among the young group of women treated by high-volume surgeons or at high-volume centers.

In addition to reduced mortality, lengths of hospital stay and hospital costs for younger women with uterine cancer were lower. The reduced rate of medical comorbidities at baseline in younger women likely contributed to this difference as preoperative comorbidities are an independent predictor of length of postoperative hospitalization and therefore cost [22]. Younger women were more likely to be managed at university-based hospitals and more likely to have an operating surgeon that was the same attending physician of record decreasing the opportunity for miscommunication and repetition of testing that could occur when multiple providers are involved in patient care. Rogers and Curtis [23] had shown that continuity of care leads to decreased hospital admissions, decreased length of stay, reduced duplication of diagnostic testing, increased patient satisfaction, and improved compliance. The high level of concordance in the younger women may account for the improved outcomes such as the decreased cost and shorter length of stay. 

The analysis suggests that younger women were less likely to be managed by high-volume surgeons. For women ≤50 years, there was a trend toward management at low-volume centers. A perceived lower risk of cancer risk or mortality, limited financial resources, unwillingness to travel long distances for care, and the absence of medical comorbidities may lead fewer younger women to seek out the expertise of a high-volume specialist on their own. Several studies have shown the benefit of women that have been cared for by a gynecologic oncologist [9, 24, 25]. Although the overall peri-operative outcomes were similar, the subspecialists were more likely to perform comprehensive surgical staging, if necessary, and the patient was less likely to receive adjuvant radiotherapy. The overall survival favored those patients managed by the general gynecologist, with no difference in disease-free interval. This may reflect the patient population that is usually managed by the general gynecologist, as compared to the gynecologic oncologist.

Similar to the differences in costs of care between younger and older women, younger women treated at low-volume settings had lower costs of care and shorter hospital stays. The reduced rate of medical comorbidities of women treated at these settings is likely to contribute to these cost differences for the same reasons as mentioned previously. The HSCRC database cannot be queried to clarify whether high-volume surgeons are located exclusively at high-volume hospitals; therefore it is unclear whether higher costs are impacted by more frequent care by gynecologic oncologists. Low-volume hospital costs may also be reduced due to the different resources of these facilities. Surgeons may be less inclined to perform aggressive procedures if these low-volume facilities do not have adequate support (i.e., access to consultants or high-acuity ICU care) and thus refer complex patients to high-volume centers. Improved efficiency at low-volume hospitals cannot be excluded as an alternative cause for lower costs.

The strength of this study lays in the relatively large number of patients included form institutions across the state of Maryland. Unfortunately, the Maryland HSCRC database is limited in ways typical of large population-based studies as it does not provide followup beyond 30 days from the index admission and contains no information on either AJCC or FIGO stage of disease, tumor grade, or histological subtype. Given these restrictions, a meaningful analysis of long-term surgical outcome was not practical from the available data. Also, the HSCRC does not provide information in terms of performance status, ASA scores, and comorbidities. These factors may have had an impact on the overall morbidity and mortality between the two populations studied. Furthermore, additional information regarding the baseline medical condition of the populations was not examined, and this could be a confounding factor on the results. A second limitation is that there is a possibility that the grouping by case volume could be too selective and there could be subgroups in the lower-volume level that still are able to achieve excellent outcomes. Due to the lack of previous reports of case volume among uterine cancer, a third limitation of the current study is that our selected volume criteria have not been widely validated as outcomes measures.

Despite these limitations, the current study provides the most extensive evaluation on volume-based care among young women that underwent surgery for uterine cancer. The information obtained in this study increases our current knowledge of volume-based care of young women diagnosed with uterine cancer and may be useful in developing strategic planning in order to improve the care offered to this population.

## Figures and Tables

**Table 1:** Demographic characteristics among women of different age groups undergoing primary surgery for uterine cancer in Maryland, 1994–2005.

Variable	≤50 years, *n* (%)	>50 years, *n* (%)
Number of patients	844 (13.6)	5337 (86.4)

Ethnic classification		
White	658 (78.0)	4205 (78.7)
African-American	125 (14.8)	937 (17.6)
American-Indian or Eskimo	34 (4.0)	132 (2.5)
Asian or Pacific Islander	23 (2.7)	42 (0.8)
Unknown	4 (0.5)	21 (0.4)

Insurance payer		
Medicaid/Medicare	67 (7.9)	2854 (53.5)
Commercial	156 (18.5)	478 (8.9)
HMO	340 (40.3)	1023 (19.2)
Blue Cross	248 (29.4)	833 (15.6)
Other	33 (3.9)	149 (2.8)

Hospital type		
Community	569 (67.4)	3532 (66.2)
Community teaching	179 (21.2)	1271 (23.8)
University	96 (11.4)	534 (10.0)

Hospital volume		
Low volume^a^	405 (48.0)	2441 (46.0)
High volume^b^	439 (52.0)	2896 (54.0)

Surgeon volume		
Low volume^c^	562 (66.6)	3296 (61.8)
High volume^d^	267 (31.6)	1874 (35.1)
Unknown	15 (1.8)	167 (3.1)

Attending physician or operating surgeon concordance		
Concordance	761 (90.2)	4622 (86.6)
Discordance	66 (7.8)	538 (10.1)
Unknown	17 (2.0)	177 (3.3)

^a^<200cases/study period.

^b^≥200 cases/study period.

^c^<100 cases/study period.

^d^≥100 cases/study period.

**Table 2:** Variables influencing age among women treated by primary surgery for uterine cancer in the state of Maryland, 1994–2005.

Covariate	≤50 years, median (range)	>50 years, median (range)	*P *value
Total adjusted cost	$6,160 ($520–185,342)	$6,587 ($0–422,661)	0.0002
ICU Days^a^	0 (0–28)	0 (0–103)	0.0800
Length of stay (days)	3 (0–46)	3 (0–126)	<0.0001

^a^ Intensive Care Unit.

**Table 3:** Variables influencing surgeon volume among women aged ≤50 years in the state of Maryland during 1994–2005.

Covariate	Low-volume surgeon, median (range)	High-volume surgeon, median (range)	*P* value
Total adjusted cost	$5,374 ($520–120,461)	$7,724 ($1598–185,342)	<0.0001
ICU Days^a^	0 (0–5)	0 (0–28)	0.2900
Length of stay (days)	3 (0–42)	3 (1–46)	0.0030

^a^ Intensive Care Unit.

**Table 4:** Variables influencing hospital volume among women aged ≤50 years in the state of Maryland during 1994–2005.

Covariate	Low-volume hospital, median (range)	High-volume hospital, median (range)	*P* value
Total adjusted cost	$5,663 ($1,455–46,702)	$6,560 ($520–8,450)	0.0100
ICU Days^a^	0 (0–5)	0 (0–28)	0.6000
Length of stay (days)	3 (0–42)	3 (0–46)	0.5400

^a^ Intensive Care Unit.
